# Ferulic acid supplementation in embryonic eggs improves heat resistance of broilers by optimizing thermal manipulation effects

**DOI:** 10.1016/j.psj.2026.107436

**Published:** 2026-07-16

**Authors:** Nawei Jike, Hanke Wang, Shixiong Lai, Yucai Sun, Yongliang Zhang, Tingting Liu, Fengzhe Chen, Zhichao Hei, Weidong Feng, Kaiqing Rao

**Affiliations:** a Key Laboratory of Animal Medicine of Sichuan Education Department, Southwest Minzu University, Chengdu, 610041, China; b Songtao Saint Laurent Agricultural Technology Company Limited in Guizhou, Tongren Guizhou, 554300, China; c Animal Husbandry Research Institute of Ganzi Prefecture, 626000, China

**Keywords:** Broiler, Embryonic thermal manipulation, Ferulic acid, Antioxidant capacity, Heat tolerance

## Abstract

Embryonic thermal manipulation (TM) enhances heat tolerance in broilers but often reduces hatchability and impairs chick quality. This study investigated the effects of TM combined with soaking embryonic eggs in ferulic acid (FA) solution on the growth performance and heat resistance of broilers. The objective was to optimize the TM regime and clarify the mechanisms by which FA-pretreated TM affects the heat tolerance in growing broilers exposed to heat stress. A total of 1000 fertilized eggs (57 ± 4 g) were randomly divided into three groups: control, TM and TM+FA (soaked in 120 mg/L FA solution from embryonic days 6 to 8). Broilers in the TM and TM + FA groups underwent thermal manipulation from embryonic day 7 to day 16 (10 days) at 39.5 °C and 65% relative humidity for 18 h daily. After hatching, the chicks were reared under standard conditions until 28 days of age, followed by a 2-week chronic cyclic heat stress challenge (35 ± 2 °C, 8 h/d). The results showed that TM+FA improved the hatchability, decreased serum corticosterone levels and feed-to-gain ratio of 28-day-old broilers, and downregulated the gene expression of thyrotropin-releasing hormone (*TRH*) in the hypothalamus, decreased body temperature of broilers under heat stress (*P* < 0.05). Both TM and TM+FA alleviated heat-stress-induced liver injury, as evidenced by enhancing antioxidant capacity and decreasing serum alanine aminotransferase activity, and inhibited cardiomyocyte apoptosis via lowering the *Bax/Bcl-*2 ratio and decreasing *HSP90* gene expression (*P* < 0.05). In conclusion, soaking TM-treated embryonic eggs in FA solution effectively improves the hatchability and heat tolerance of broilers during the post-hatch growth period. Thus, the TM + FA treatment provides a more reliable approach than the single TM treatment to combating heat stress in poultry production.

## Introduction

Broilers are covered with feathers and lack sweat glands, rendering them inherently poor at heat tolerance. The optimal environmental temperature range for broilers is 21 ∼ 26 °C. When the temperature rises to 26 ∼ 32 °C, broilers may feel discomfort, but can still maintain normal physiological function (De et al., 2024). However, when the ambient temperature is higher than 32 °C, broilers suffer from heat stress (HS) (Brugaletta et al., 2022), resulting in physiological dysfunction. Moreover, increased stocking density and high humidity can exacerbate the severity of HS (Wan et al., 2023). High temperatures adversely affect poultry behavior and physiology, such as decreased feed intake, increased water intake, depressed spirits, and panting. It also disrupts the body's oxidative balance in chickens, activates the hypothalamic-pituitary-adrenal (HPA) stress axis, damages the respiratory tract, digestive tract, and immune function, and ultimately impairs growth performance in broilers (Campos et al., 2025). Given the increasingly severe challenges posed by extreme weather, protecting broilers from high temperature stress, mitigating the damage of HS to the body, and reducing the economic losses of breeding has become an urgent theoretical and practical problem to be solved (Hosseinzadeh et al., 2026).

Exposure to elevated ambient temperatures induces functional modifications in the neuroendocrine system of avian species. This physiological response triggers HPA axis activation, consequently increasing plasma corticosterone (CORT) concentrations (Madison et al., 2008). Puvadolpirod and Thaxton (2000) reported that the stress response in broiler chickens includes increases in plasma CORT, glucose, cholesterol, triglycerides, high-density lipoprotein (HDL) and total protein. Tang et al. (2022) found that aspartate aminotransferase (AST) activity was increased when broilers were continuously exposed to HS (32°C) for 1 or 2 weeks. The observed elevations in serum alanine aminotransferase (ALT) and AST levels correlate with progressive liver injury, driven by endoplasmic reticulum stress and cellular apoptosis caused by high temperature (Ma et al., 2020). Oxidative injury in broilers resulting from high thermal conditions has been documented by multiple investigations (Lin et al., 2008; Mujahid et al., 2007). Research indicates that heat stress triggers a thermal adaptation response in poultry, leading to marked upregulation of both heat shock proteins (*HSP*) *90α* mRNA and *HSP90β* mRNA in cardiac, hepatic, and splenic tissues (Abare et al., 2023). Elevated tissue *HSP* mRNA expression implies these proteins may provide protection under adverse conditions, facilitating animal adaptation to environmental stressors.

Thermal manipulation (TM) refers to temporarily increasing the incubation temperature (e.g., 39.5 °C) during mid-embryogenesis (embryonic days 7-16) to induce thermal adaptation. Piestun et al. (2008a, 2008b, 2011, 2015) demonstrated that both intermittent TM (12 h/d, 18 h/d) and continuous TM (24 h/d) applied from E7 to E16 significantly reduced post-hatch body temperature compared with untreated controls. A separate investigation exposed embryonic eggs to 39.5°C and 65% RH for 3 hours daily during mid- (E8 - E10) and late-stage (E16 - E18) incubation. This regimen also resulted in a significantly lower body temperature in the treated broilers compared with the control group (Alkan et al., 2012). The decreased body temperature observed in these experiments indicates that embryonic TM induces changes in the hypothalamic-pituitary-thyroid (HPT) and HPA axes, which directly leads to a downward shift in the body temperature set point. Consequently, it enhances the heat tolerance and improves stress adaptation in broiler chickens after hatching (Al-Zghoul et al., 2025).

Embryonic TM can improve the chicken's heat resistance, however, to achieve this effect, the TM duration needs to be 12 h/d or longer, which consequently reduces the hatchability and chick quality (Zaboli et al., 2017). Hatchability represents the percentage of fertilized eggs that successfully hatch. The healthy chick rate reflects the proportion of newly hatched chicks without umbilical infection, normal standing posture and good vitality. Recent studies have suggested that thermal manipulation during embryogenesis induces epigenetic metabolic adaptations, enhancing the embryo's ability to cope with high ambient temperatures (Al Amaz and Mishra, 2024). FA is a phenolic acid ubiquitous in nature and has multiple biological functions, acting as both an antioxidant and an anti-inflammatory factor (Kumar et al., 2025). This substance actively eliminates oxidative radicals, reduces the formation of reactive oxygen species (ROS), and participates in a variety of signal transduction pathways to exert its antioxidant effect and mitigate inflammatory responses (Zduńska et al., 2018). Earlier work from our laboratory established that immersing broiler hatching eggs in ferulic acid solution during incubation stimulates the development of the embryonic allantoic membrane and strengthens antioxidant defenses in both embryos and newly hatched chicks (Lai et al., 2022). Additionally, FA can effectively protect TM-exposed chicken embryos by regulating the Nrf2 antioxidant signaling pathway in the liver (Lai et al., 2024). The efficacy of FA solution immersion for TM-exposed embryonic eggs in modulating post-hatch heat adaptation of broilers requires further investigation. Based on previous findings from our laboratory, the optimal concentration of FA solution was determined to be 120 mg/L, which was consequently selected for our present study (Chen et al., 2025).

Heat stress increases reactive oxygen species (ROS) production and triggers oxidative stress in broilers, ultimately causing oxidative damage to cellular lipids, proteins, and DNA (Mujahid et al., 2007). The liver, a core organ governing systemic metabolism, is responsible for nutrient metabolism, energy storage, and detoxification (Robinson et al., 2016). Under high-temperature exposure, elevated serum ALT and AST activities are reliable markers of liver injury, which is closely linked to endoplasmic reticulum stress and cellular apoptosis (Ma et al., 2020). Accordingly, assessments of antioxidant status and liver function are critical for evaluating the protective effects of TM combined with FA against heat stress in broilers.

Although previous studies have demonstrated that embryonic TM can enhance post-hatch heat tolerance in broilers, its practical application is hindered by reduced hatchability and impaired chick quality. Furthermore, it remains unclear whether antioxidant supplementation, such as FA, can mitigate these adverse outcomes while maintaining or even improving the heat resistance benefits of TM. To date, the combined effects of FA soaking and TM on post-hatch growth performance, antioxidant capacity, and the expression of heat stress-related genes have not been systematically investigated.

The primary objective of the current investigation was to identify effective countermeasures against the detrimental impacts of embryonic thermal manipulation—specifically, reduced hatchability and impaired chick quality—by investigating whether pre-treatment with the antioxidant FA can mitigate these drawbacks. Furthermore, we aimed to explore the effects of TM alone versus TM combined with FA on heat resistance in broilers, as well as the underlying mechanisms. This was achieved by examining changes in antioxidant capacity, serum biochemical indices, cardiac cell apoptosis, and the expression of *HSP* genes, thereby providing a theoretical basis for optimizing the application of TM with FA supplementation to combat heat stress in broiler production.

## Materials and methods

### Animal ethics

This study was approved by the Animals Care and Ethics Committee of Southwest Minzu University (Approval code:SMU-202501156).

### Experimental design

A total of 1,000 fertilized eggs from yellow-feather broilers, weighing 57 ± 4 g, were randomly divided into three groups: control group (n = 380), TM group (n = 350) and TM+FA group (n = 270). All experimental eggs originated from 45-week-old laying hens. The number of hatching eggs in each group was determined based on the hatchability observed in a preliminary experiment. To maintain consistent group sizes for the subsequent post-hatch heat stress challenge (in which the control group was split into a negative control group and a heat-stress group), a larger initial number of eggs was allocated to both the control and TM groups than to the TM+FA group. This design compensated for the expected decrease in hatchability caused by embryonic TM exposure—an effect that was counteracted by FA supplementation. After sterilization, the eggs were incubated in three incubators of the same model from the same manufacturer (KFK microcomputer automatic incubator, Keyu Incubation Equipment Co., LTD.). Standard incubation parameters (37.8°C, 56% RH, automatic egg turning every 2 h) were maintained consistently for the control group eggs throughout the experimental period. From the 7th to16th day of incubation, the eggs of the TM and TM+FA were exposed to a higher temperature of 39.5°C with 65% RH for 18 hours daily, with automatic egg turning every 2 h. At other times, their incubation conditions were the same as those of the control group. The surface temperature of the eggshell was measured twice daily using an infrared thermometer (DT-8838, CIM-Huacheng) to ensure that the eggs maintained the set temperature of the incubator.

An aqueous FA solution (120 mg/L) was prepared under dark conditions. For preparation, 720 mg of FA powder (purity > 99%; Shanghai Yuanye Biotechnology Co., Ltd., China) was dissolved in 6 L of purified water maintained at 60°C. Complete dissolution was achieved by continuous stirring with a glass rod. From embryonic days 6 to 8, eggs in the TM+FA group underwent daily immersion in FA solution at 38°C for 6 minutes at 18:00. After immersion, the eggs were removed, placed on the hatching plate, and returned to the incubator after the eggs were dry (the environmental temperature was set to 30°C). The specific conditions for TM treatment and the FA immersion methods for embryonic eggs were based on previous findings from our laboratory (Lai et al., 2024).

When the eggs had hatched between embryonic days 20 and 21, all chicks were removed from the incubator, weighed, and placed in an environment maintained at 35 °C. Weak chicks displaying defined criteria—including umbilical roughness, severe debilitation, and postural instability—were systematically counted across all experimental groups. After hatching, the unhatched eggs were opened to count the number of unfertilized eggs and dead embryos in each group. Subsequently, the hatchability, healthy chick rate and hatchling weight were calculated. All chicks proceeded to subsequent rearing phases in accordance with their original experimental group assignments. Chicks had *ad libitum* access to food and water, with a 24-hour light cycle. The feeding method was floor rearing with bedding material, with approximately 10 cm of rice husks placed on the ground. There were three groups, with 6 pens per group (18 pens in total). Each replicate corresponded to one pen, measuring 1.6 m × 0.75 m (approximately 1.2 m^2^).

At 28 days of age, 480 healthy chicks (equal numbers of males and females) were selected and randomly assigned to four groups for subsequent rearing: control group, heat stress group, TM+HS group, and TM+FA+HS group. Specifically, a total of 240 healthy chicks were selected from the original control group and randomly allocated into the control group and the heat stress group. A total of 120 chicks were selected from the TM group to form the TM+HS group, and another 120 chicks were selected from the TM+FA group to form the current TM+FA+HS group. Thus, four groups were set up, with six replicates per group and 20 chickens per replicate, yielding in 24 pens in total.

Heat stress treatment was implemented between 29 and 42 days of age, and relative humidity was controlled at 60% ∼ 70%. Control broilers were housed at 25 ± 2°C (regulated by air conditioning) with natural nighttime temperature variations, while the other groups received daily 8-hour heat exposure (35°C from 10:00 am to 6:00 pm) using a fuel-powered heating system, after which the temperature returned to baseline (25°C ∼ 27°C) within 1 hour. All birds had free access to feed and water for the duration of the study. At weekly intervals, residual feed within each pen and the body weight of chicks were meticulously recorded. Based on these records, average daily gain, daily feed intake, and feed conversion ratio were subsequently determined.

## Measurements

### Growth performance

On the first day, each chick was weighed and allocated to its specific pen. Feed intake was recorded starting on day 3, as the diet was provided in a wet form during the first three days after hatching.The body weight and feed consumption for each pen were monitored weekly on days 3, 7, 14, 21, 28, 35, and 42. From these weekly records, key performance metrics—including average daily gain (ADG), average daily feed intake (ADFI), and feed conversion ratio (FCR)—were derived for two periods: pre–heat stress (3 d ∼ 28 d) and heat stress (29 d ∼ 42 d).

### Body temperature

At 3, 7, 14, 21 and 28 days of age, three chickens were randomly selected from each replicate of each group. The probe of an electronic thermometer was coated with medical Vaseline and then inserted into the chickens' anus to measure rectal temperature. On days 1, 3, 5, 8, 10, 12 and 14 of HS treatment, three chickens were randomly selected from each replicate of each group. An ADT-8838 professional infrared thermometer was used to quickly measure the temperature under the broilers' wings.

### Sample collection

At 28 days of age, 8 chickens from each group were randomly selected for blood sampling via the wing vein, and the serum was separated and stored at - 80 °C. Upon conclusion of the 42-day trial, two chickens from each replicate pen replicate pen (equivalent to 12 birds per treatment) were euthanized using carbon dioxide gas. These birds were then processed for tissue and biological sample collection. The serum was separated, and the brain, heart, liver, spleen, and bursa of Fabricius were collected for weighing and organ index calculation. Hypothalamic, cardiac, and hepatic tissues were collected, immediately snap-frozen in liquid nitrogen, and maintained at −80 °C for subsequent analysis.

The organ index was calculated as follows: Organ index (%) = (organ weight / body weight) × 100.

### Preparation and HE staining of liver sections

The fixed liver tissues from each group were embedded in conventional paraffin wax. The wax blocks were then sectioned, deparaffinized, hydrated, stained with hematoxylin-eosin (HE) and mounted as liver tissue slides. Images were collected using a ZEISS laser confocal microscope, with 10× and 40× magnification fields captured from each group of sections.

### Determination of antioxidant properties

Eight serum samples from 28-day-old broilers were randomly selected from each group, while 12 serum, heart and liver samples from 42-day-old broilers were randomly selected from each group to determine the activities of SOD (A001-3-2), CAT (A007-1-1) and GSH-Px (A005-1-2), as well as MDA (A003-1-2) contents. The kits used to measure these oxidation-related indicators were purchased from Nanjing Jiancheng Bioengineering Institute, and the procedures were performed according to the manufacturer's instructions. Cardiac catalase activity fell below the detection threshold of the assay kit in all samples, which aligns with the tissue-specific antioxidant defense mechanisms of myocardial tissue; consequently, these data were excluded from subsequent statistical analysis.

### Detection of serum hormones by ELISA

The levels of CORT, triiodothyronine (T_3_) and tetraiodothyronine (T_4_) in serum from 28-day-old and 42-day-old broilers were detected using ELISA kits based on the competitive method. The chicken CORT (E-OSEL-Ch0002) kits were purchased from Wuhan Ilerit Biotechnology Co., Ltd., while the T_3_ (H222-1-2) and T_4_ (H223-1-2) kits were obtained from Nanjing Jiancheng Bioengineering Institute.

### Determination of serum liver function-related indices

Twelve serum samples from 42-day-old broilers were randomly selected. The activities of AST (C010-1-1) and ALT (C009-1-1) in these samples were measured using a colorimetric assay. All assay kits were purchased from Nanjing Jiancheng Bioengineering Institute, and the experimental procedures were conducted strictly in accordance with the manufacturer's instructions.

### Quantitative real-time PCR

Following the manufacturer's protocol, total RNA was extracted from broiler hypothalamic and cardiac tissues using the RNA isolator Total RNA Extraction Reagent (Vazyme Biotech Co., Ltd., Nanjing). Six biological replicates were included per experimental group. RNA purity (OD260 nm/OD280 nm ratio) and concentration were measured with an ultra-micro nucleic acid/protein analyzer (NanoDrop ONEC, Anhui Hongzhong Medical Equipment Co., Ltd.). RNA quality and integrity were assessed by denaturing electrophoresis (EPS-600, Shanghai Nashi Biotechnology Co., Ltd.). Subsequently, cDNA templates were synthesized from the prepared samples using the ABScript Ⅲ RT Master Mix for qPCR with gDNA Remover (RK20429) on a PCR instrument (TY2021006095). Primer sequences for the *β-actin, HSP60, HSP70, HSP90, Bax, Bcl-2, TRH*, and *CRH* genes (Table 1), obtained from previously published literature, were synthesized by Sangon Biotech (Shanghai) Co., Ltd. Quantitative real-time PCR was performed using the 2×Universal SYBR Green Fast qPCR Mix (ABclonal RK21203) according to the manufacturer's instructions on a fluorescence quantitative PCR system (Thermo QuantStudio 3, Thermo Fisher Scientific (China) Co., Ltd.). The specificity of amplification was verified by melting curve analysis, which produced a single peak for each amplicon. The PCR amplification efficiencies for all primer pairs, as determined by standard curve analysis, ranged from 90% to 110%. The relative expression of each mRNA was calculated using the 2^−△△Ct^ method, with *β-actin* gene expression used for normalization.

**Table 1:** Primers for qPCR.

Genes	GenBank accession No.	Primer sequences
*β-actin*	NM_205518.2	F: TGCGTGACATCAAGGAGAG
		R: TGCCAGGGTACATTGTGGTA
*HSP60*	NM_001012916.3	F: AGCCAAAGGGCAGAAATG
		R: TACAGCAACAACCTGAAGACC
*HSP70*	NM_001006685.2	F: CTGGCAATAAGCGAGCAGTGAGG
		R: AATGCTGGCTTGCGTGGAAGAG
*HSP90*	NM_001109785.2	F: TCCTGTCCTCTGGCTTTAGTTT
		R: AGGTGGCATCTCCTCGGT
*Bax*	XM_046922136.1	F: GTGATGGCATGGGACATAGCTC
		R: TGGCGTAGACCTTGCGGATAA
*Bcl-2*	XM_046932962.1	F: ACCCGGAGATCATGGAGA
		R: GATGCCTTGCTGGTAGACG
*CRH*	NM_001123031.1	F: CATCTCCCTGGACCTGACTTTC
		R: TCCTGTTGCTGTGGGCTTG
*TRH*	NM_001030383.2	F: AGCCACATGCTTCAATCT
		R: TCCAGGTAGTTGACAAGGTA

### Statistical analysis

Experimental data are presented as the mean ± standard error of the mean (SEM). All data were first assessed for normality using the Shapiro-Wilk test and for homogeneity of variance using Levene's test. Normally distributed data were analyzed by one-way ANOVA followed by Tukey's post hoc test. Hatchability and healthy chick rates were analyzed using the Chi-square test. Differences were recognized significant at *P* ≤ 0.05, and statistical trends were recognized at 0.05 < *P* < 0.1*.*

## Results

### The hatching performance of fertilized eggs

Table 2 shows that compared with the control group, hatchability, healthy chick rate, and chick weight in the TM and TM+FA groups were significantly lower (*P* < 0.05). Compared with the TM group, hatchability in the TM+FA group was significantly higher (*P* < 0.05). Additionally, compared with the TM group, the TM+FA group showed a 5% increase in the healthy chick rate.

**Table 2:** Effects of thermal manipulation and ferulic acid soaking on hatchability, chick body weight, and healthy chick rate.

Groups	Chick weight(g)	Hatchability(%)	Healthy chick rate(%)
Control	40.47 ± 0.15^a^	92.78^a^	86.49^a^
TM	39.91 ± 0.19^b^	69.91^c^	65.82^b^
TM+FA	39.84 ± 0.18^b^	85.77^b^	70.85^b^

Data within a common column marked with distinct lowercase superscript letters indicate statistical significance (*P* < 0.05). Conversely, entries sharing identical superscript letters or lacking such notations demonstrate no significant difference (*P* > 0.05). This convention applies to all subsequent tables.

### Productive performances

Table 3 shows that from 3 to 28 days of age, compared with the control group, ADFI and ADG in the TM and TM+FA groups were significantly decreased (*P* < 0.05), and the FCR of broilers in the TM + FA group was significantly lower than that in the control group (*P* < 0.05). From 29 to 42 days of age, there were no significant differences in ADFI, ADG or FCR between the TM or TM+FA groups and the control group (*P* > 0.05).

**Table 3:** Effects of thermal manipulation and ferulic acid soaking on broiler post-hatch growth performance.

Groups		Control	TM	TM+FA
3 ∼ 28 d	ADFI/(g)	45.28 ± 0.31^a^	38.56 ± 0.51^b^	38.35 ± 0.57^b^
	ADG/(g)	25.24 ± 0.20^a^	22.35 ± 0.65^b^	22.45 ± 0.31^b^
	FCR	1.80 ± 0.01^a^	1.73 ± 0.032^ab^	1.71 ± 0.034^b^
29 ∼ 42 d (heat stress)	ADFI/(g)	70.16 ± 2.26	64.14 ± 3.16	68.80 ± 0.82
	ADG/(g)	29.65 ± 1.94	25.11 ± 0.57	28.38 ± 2.10
	FCR	2.42 ± 0.18	2.55 ± 0.09	2.49 ± 0.17

^a,b^ Means with different superscripts within a column are different at *P* < 0.05.

### Measurement of broiler body temperature

**Fig. 1:**
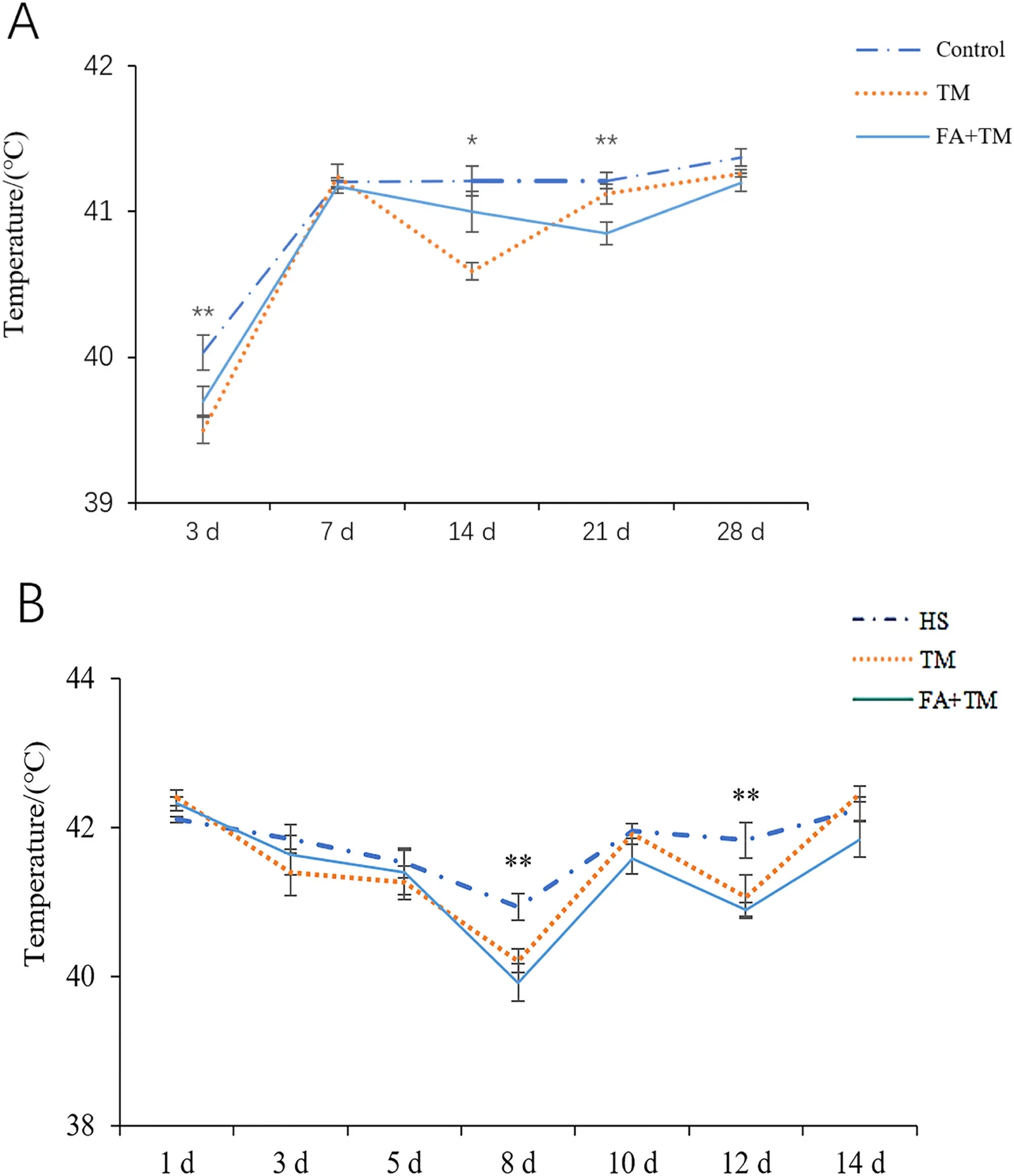
Effects of thermal manipulation and ferulic acid soaking on broiler body temperature. (A) Broiler body temperature at 1 to 28 days of age. (B) Broiler body temperature under heat stress (28 to 42 days of age). Data are presented as means ± SEM. * means *P* < 0.05, ** means *P* < 0.01 compared with the control group.

The results show that compared with the control group, the body temperature in the TM group was significantly lower at 3 and 14 days of age (*P* < 0.05), and the TM+FA significantly reduced broiler body temperature at 3 and 21 days of age (*P* < 0.05) (Fig. 1A). During the heat stress period, on days 8 and 12, the broiler body temperature in both the TM and TM+FA groups was significantly lower than that in the control group (*P* < 0.05) (Fig. 1B).

### Organ indices of broilers at 42 days of age

As shown in Table 4, the liver index of the TM+FA+HS group was significantly higher than that of the HS group (*P* < 0.05). No significant difference was observed among groups in heart index, spleen index, brain index or bursa of Fabricius index (*P* > 0.05).

**Table 4:** Effects of thermal manipulation and ferulic acid soaking on broiler organ indices.

Groups	Heart (mg/g)	Liver (mg/g)	Spleen (mg/g)	Brain (mg/g)	Bursa of Fabricius (mg/g)
HS	3.74 ± 0.15	27.03 ± 1.07^b^	2.05 ± 0.19	1.75 ± 0.070	1.11 ± 0.13
TM+HS	3.97 ± 0.17	30.64 ± 1.15^ab^	1.87 ± 0.16	1.87 ± 0.071	1.25 ± 0.21
TM+FA+HS	3.70 ± 0.10	31.82 ± 1.28^a^	1.75 ± 0.10	1.86 ± 0.062	1.24 ± 0.16

^a,b^ Means with different superscripts within a column are different at *P* < 0.05.

### Serum hormone levels at 28 and 42 days of age

Table 5 shows that at 28 days of age, the TM+FA treatment significantly reduced serum CORT levels in broilers (*P* < 0.05), while the T_3_ level showed a downward trend (0.05 < *P* < 0.1). TM significantly reduced serum T_3_ levels (*P* < 0.05), with the CORT levels also showing a downward trend (0.05 < *P* < 0.1). Serum T_4_ levels in the TM group and TM+FA group were not significantly different from those in control group (*P* > 0.05). At 42 days of age, the serum T_4_ level in the TM+HS group was lower than that in the HS and TM+FA+HS group (0.05 < *P* < 0.1). Meanwhile, neither TM+HS nor TM+FA+HS had a significant effect on serum CORT or T_3_ levels in broiler under HS (*P* > 0.05).

**Table 5:** Effects of thermal manipulation and ferulic acid soaking on broiler serum hormone levels.

	Groups	CORT/(ng/mL)	T_3_/(ng/mL)	T_4_/(ng/mL)
28 d	Control	174.31 ± 14.20^a^	3.56 ± 0.22^a^	93.22 ± 9.38
	TM	137.08 ± 12.19^ab*^	2.85 ± 0.15^b^	88.46 ± 6.71
	TM+FA	134.89 ± 9.84^b^	3.00 ± 0.25^ab*^	79.56 ± 8.14
42 d	HS	182.84 ± 24.10	1.88 ± 0.10	99.76 ± 3.49
	TM+HS	217.05 ± 17.95	2.04 ± 0.11	84.41 ± 5.46^*$^
	TM+FA+HS	208.74 ± 9.27	1.98 ± 0.09	98.64 ± 6.81

^a,b^ Means with different superscripts within a column are different at *P* < 0.05.

In this table, "*" indicates an upward or downward trend compared to the control group (0.05 < *P* < 0.1), and "$" indicates an upward or downward trend compared to the TM+FA group (0.05 < *P* < 0.1).

### The antioxidant capacity of chicks at 28 and 42 days of age

As shown in Table 6, at 28 days of age, TM significantly increased the serum SOD and GSH-Px activities in broiler compared with the control group (*P* < 0.05). In the TM+FA group, serum SOD activity was significantly increased (*P* < 0.05), and MDA content was significantly decreased (*P* < 0.05). After HS, compared with the HS group, serum GSH-Px activity was significantly increased in both the TM+HS group and TM+FA+HS group (*P* < 0.05). Additionally, liver MDA content in these two groups was significantly lower than that in the HS group (*P* < 0.05). The TM+FA+HS treatment significantly increased liver CAT activity, as well as heart SOD and GSH-Px activities (*P* < 0.05). Cardiac CAT activity was undetectable and was therefore excluded from the results.

**Table 6:** Effects of thermal manipulation and ferulic acid soaking on broiler serum antioxidant related indices.

Days	Groups	MDA (nmol/mL)	CAT (U/mL)	SOD (U/mL)	GSH-Px (U/mL)
Serum of 28 d broilers	Control	2.33 ± 0.18^a^	2.07 ± 0.27	16.55 ± 0.50^b^	320.36 ± 7.68^b^
	TM	2.01 ± 0.13^a^	1.95 ± 0.20	19.83 ± 1.08^a^	344.04 ± 6.52^a^
	TM+FA	1.50 ± 0.11^b^	1.57 ± 0.11	19.35 ± 1.21^a^	330.11 ± 5.40^ab^
Serum of 42 d broilers	HS	2.44 ± 0.38^ab^	2.04 ± 0.26	32.62 ± 1.93	403.88 ± 10.16^b^
	TM+HS	2.77 ± 0.36^a^	2.08 ± 0.28	34.83 ± 1.81	443.33 ± 5.94^a^
	TM+FA+HS	1.64 ± 0.16^b^	1.88 ± 0.22	30.43 ± 1.56	439.40 ± 4.73^a^
Liver of 42 d broilers	HS	0.33 ± 0.018^a^	12.72 ± 1.34^b^	52.68 ± 1.84	28.28 ± 2.20
	TM+HS	0.27 ± 0.012^b^	10.23 ± 1.10^b^	55.55 ± 1.71	25.69 ± 1.80
	TM+FA+HS	0.27 ± 0.011^b^	14.90 ± 1.57^a^	54.44 ± 1.92	26.90 ± 2.08
Heart of 42 d broilers	HS	0.42 ± 0.032	–	51.80 ± 3.57^ab^	2.82 ± 0.25^b^
	TM+HS	0.40 ± 0.024	–	42.05 ± 3.39^b^	3.51 ± 0.35^ab^
	TM+FA+HS	0.40 ± 0.016	–	54.50 ± 4.11^a^	3.73 ± 0.40^a^

^a,b^ Means with different superscripts within a column are different at *P* < 0.05.

### The liver HE-stained slices and the activity of serum AST and ALT at 42 days of age

**Fig. 2:**
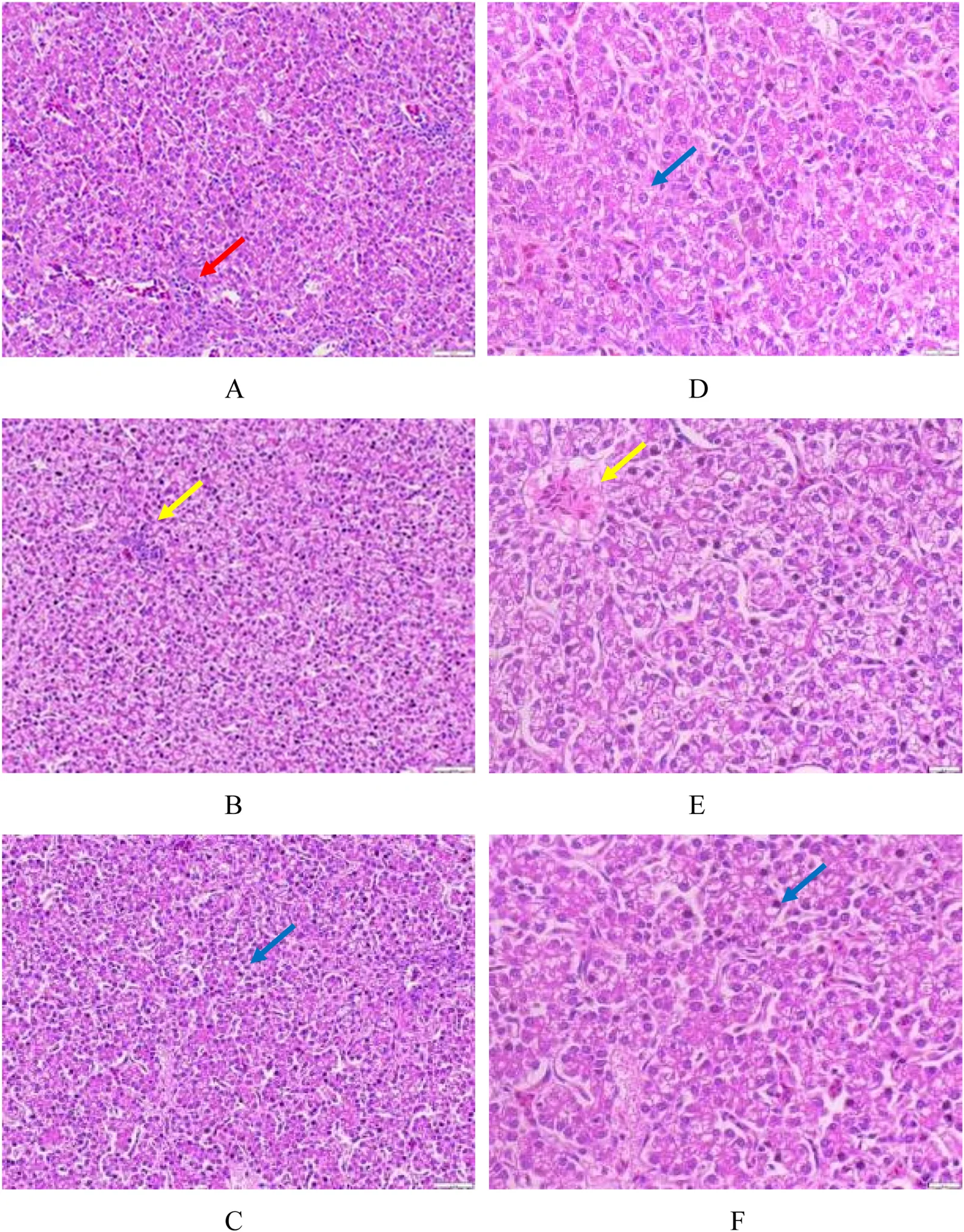
Effects of thermal manipulation and ferulic acid soaking on broiler liver histology under heat stress (HE staining). (A, D) HS group; (B, E) TM+HS group; (C, F) TM+FA+HS group. Magnification: 10×(A-C) and 40×(D-F). Blue arrow indicates edematous hepatocytes, red arrow indicates lymphocyte infiltration, yellow arrow indicates normal red blood cells in the central vein.

As shown in Fig. 2, the liver tissue structure was disorganized in the HS group, the TM+HS group and the TM+FA+HS group (Panels A, B, and C), with lymphocyte infiltration occasionally observed in Panel A (red arrow). Numerous hepatocytes were observed in panels D and F, whereas hepatocytes in panel E exhibited edema, cell swelling, and loose, lightly stained cytoplasm (blue arrows). Table 7 shows that compared with the HS group, the serum AST and ALT levels in the TM+HS group were significantly lower (*P* < 0.05). Additionally, the ALT level in the TM+FA+HS group was significantly lower than that in the HS group (*P* < 0.05).

**Table 7:** Effects of thermal manipulation and ferulic acid soaking on broiler serum liver function indices under heat stress.

Groups	AST/(U/mL)	ALT/(U/mL)
HS	44.72 ± 3.33^a^	12.74 ± 0.78^a^
TM+HS	36.28 ± 2.08^b^	3.74 ± 0.51^c^
TM+FA+HS	40.73 ± 2.04^ab^	9.38 ± 1.09^b^

^a,b,c^ Means with different superscripts within a column are different at *P* < 0.05.

### The gene expression in hypothalamus and heart of chicks at 42 days of age

As shown in Table 8, compared with the HS group, the TM+HS and TM+FA+HS groups significantly down-regulated the gene expression of *Bax* and *HSP90* in broiler heart (*P* < 0.05). The *Bax/Bcl-2* ratio was significantly lower in the TM+FA+HS group than in the HS group (*P* < 0.05), and the gene expression of *HSP70* was significantly higher in the TM+FA+HS group than that in the TM+HS group (*P* < 0.05). However, no significant differences were observed among the groups in the gene expression of *Bcl-2* or *HSP60* (*P* > 0.05). As shown in Table 9, *TRH* gene expression in the hypothalamus was significantly lower in the TM+FA+HS group than that in HS group and TM group (*P* < 0.05).

**Table 8:** Effects of thermal manipulation and ferulic acid soaking on cardiac apoptosis and heat shock protein gene expression in broilers under heat stress.

Groups	*Bax*	*Bcl-2*	*Bax/Bcl-2*	*HSP60*	*HSP70*	*HSP90*
HS	1.00 ± 0.08^a^	1.00 ± 0.12	1.00 ± 0.07^a^	1.00 ± 0.13	1.00 ± 0.12^b^	1.00 ± 0.16^a^
TM+HS	0.67 ± 0.08^b^	0.73 ± 0.10	0.90 ± 0.09^ab^	0.73 ± 0.12	0.82 ± 0.20^b^	0.33 ± 0.11^b^
TM+FA+HS	0.73 ± 0.09^b^	1.06 ± 0.16	0.73 ± 0.10^b^	0.98 ± 0.15	1.58 ± 0.24^a^	0.46 ± 0.09^b^

^a,b^ Means with different superscripts within a column are different at *P* < 0.05.

**Table 9:** Effects of thermal manipulation and ferulic acid soaking on *TRH* and *CRH* gene expression in broiler hypothalamus under heat stress.

Groups	*CRH*	*TRH*
HS	1.00 ± 0.12	1.00 ± 0.17^a^
TM+HS	0.94 ± 0.18	0.89 ± 0.19^a^
TM+FA+HS	1.00 ± 0.11	0.36 ± 0.07^b^

^a,b^ Means with different superscripts within a column are different at *P* < 0.05.

## Discussion

Cong et al. (2023) found that TM during embryonic development leads to decreased hatchability and healthy chick rate. Our study similarly found that TM reduced the hatchability, healthy chick rate, and hatchling weight, which is consistent with previous findings and indicates that embryonic TM actually causes damage to chicken embryos and directly affects the hatchability. Compared with TM, soaking TM embryonic eggs in FA solution significantly improved the hatchability, which suggests that FA soaking may effectively protect TM broiler embryos. Furthermore, FA might alleviate TM-induced embryonic oxidative damage by enhancing the antioxidant capacity of broilers. In the study by Lai et al. (2022), soaking broiler embryonic eggs in FA solution enhanced the development of the CAM, an effect mediated by upregulation of vascular endothelial growth factor gene expression, which concurrently boosted antioxidant capacity in chicks. Chowdhury et al. (2019) confirmed that FA (50 mg/kg) significantly reduced free radical production, inhibited the increase in intracellular ROS levels, and regulated changes in oxidative stress indicators (SOD, MDA, CAT) in diabetic rats after 8 weeks of administration. Based on existing literature, ferulic acid has been demonstrated to enhance the transcription and expression of antioxidant enzymes by facilitating the translocation of Nrf2 from the cytoplasm to the nucleus (Hu et al., 2025).

A novel observation of this study was that chicks in both TM groups exhibited significantly reduced ADFI and weight gain compared with the control group throughout the 1 - 28 day rearing period. This finding has not been reported in earlier studies (Piestun et al., 2013) and may be attributed to differences in broiler breeds. Furthermore, thermal manipulation during embryogenesis negatively affects early post-hatch growth, which results in a trade-off between programmed embryonic thermotolerance and intrinsic growth capacity. Broilers receiving FA supplementation demonstrated markedly improved feed conversion efficiency compared with the control group, presenting an interesting avenue for further investigation. Meanwhile, FA may exert a protective effect against TM-induced oxidative stress in embryos by enhancing the overall antioxidant status of the broilers. Embryonic TM decreases body temperature of broilers, reduces energy used for temperature regulation, and lowers basal metabolism. This helps broilers tolerate heat stress better in actual production. In addition, the lower feed intake in TM groups may be caused by reduced metabolic energy needs, and this correlation needs further long-term study. Embryonic TM or TM plus FA treatment reduced broiler body temperature and exhibited enhanced thermoregulation and antioxidant capacity, yet these physiological advantages did not translate into improved growth performance or feed efficiency under heat stress. On the one hand, the heat stress in this experiment was relatively severe. According to the standard Temperature-Humidity Index (THI) calculation formula (NRC, 1971), the THI value in this study reached 87, belonging to extreme heat stress level (Zulovich and DeShazer, 1990). Under severe HS, broilers consumed large amounts of energy to dissipate heat. Such physiological advantages could not counteract the inhibitory effects of intense stress on feed intake and nutrient utilization, thus failing to improve growth traits. On the other hand, physiological indices and growth performance respond to stress at different speeds. Blood and antioxidant indicators change quickly, while body weight and feed efficiency are long-term cumulative traits. Within the 14-day stress period, physiological advantages cannot immediately reflect on production performance. Such time-delayed responses have been reported in poultry heat stress studies (Attia et al., 2018; Musa et al., 2025). Subsequent trials with milder stress and longer observation duration are needed to verify our findings.

The indices for evaluating heat resistance in broilers include body temperature, blood physiology and biochemical parameters, and growth performance; among which heat resistance is primarily reflected by change in body temperature. Body temperature changes can comprehensively reflect their ability to maintain thermal balance by regulating heat production and heat dissipation, and has been widely used as an indicator of thermoregulatory capacity under heat stress conditions (Hossain et al., 2025). The optimal incubation temperature for fertilized eggs is 37 °C ∼ 38 °C (Bruzual et al., 2000). Piestun et al. (2009) found that both intermittent TM and continuous TM could significantly reduce the plasma T_3_ and T_4_ levels in broilers, with the plasma T_4_ levels being significantly lower in continuously heat-treated broilers than in intermittently heat-treated ones. This suggests that different TM durations exert different effects on enhancing the heat resistance in broilers. In this study, broilers in both the TM and TM+FA groups exhibited lower body temperature during the early rearing period and during the late HS stage. At 28 days of age, serum CORT and T_3_ levels were decreased in both treatment groups; and at 42 days of age, serum T_4_ levels in the TM group showed a decreasing trend. These results indicate that TM alters broiler metabolic levels regardless of HS exposure. Furthermore, the downregulated expression of hypothalamic *TRH* mRNA in the TM+FA+HS group suggests that TM may affect the HPT axis in broilers.

However, whether changes in metabolic levels are directly correlated with tissue damage in broilers remains to be clarified. To explore this, we measured the organ indices and other related indicators. Studies have shown that the heart, liver and kidneys are often the first organs damaged in animals under HS (Wu, 2022). Under heat stress, cardiac cells experience sustained oxidative damage to DNA and inhibition of the activity of critical metabolic enzymes involved in protein and lipid processing, accompanied by a marked decline in the activity of major antioxidant enzymes, including SOD and GSH-Px. Therefore, high-temperatures-induced oxidative stress occurs in animal heart, and cardiomyocyte death may even ensue (Wu, 2022). In this study, the liver index was significantly higher in the TM+FA+HS group than in the control group. This was accompanied by substantially higher serum AST and ALT activities in the HS group than in either treatment group, and coincided with more pronounced liver injury, as confirmed by histological examination. These observations raise the possibility that TM combined with FA may influence cellular antioxidant capacity and apoptosis. However, further work is needed to confirm any direct causal relationships.

HS induces oxidative stress in broilers, which subsequently impairs multiple physiological functions, ultimately leading to deterioration of meat quality, increased morbidity and mortality, and reduced production performance (Prates, 2025). The value of various antioxidant-rich phytochemicals, particularly a range of flavonoid compounds, has been recognized in prior research for alleviating chronic heat stress in poultry, suggesting their considerable utility in addressing this challenge (Lee et al., 2018). Sujath et al. (2010) found the 100 mg/kg extract from dried *forsythia* fruit had a stronger ability to reduce oxidative damage caused by HS in broilers than 200 mg/kg vitamin C supplementation. Research by Liu et al. (2014) revealed that resveratrol exerts differential regulation of *HSP* genes in broilers, normalizing HS-induced over-expression in the bursa and spleen while concurrently upregulating suppressed *HSP70* and *HSP90* expression in the thymus; it also increased serum antioxidant enzymes activity. These results suggest that the use of plant antioxidants in summer livestock breeding provides a strategy for broilers to combat HS. In addition to the improved antioxidant status (elevated enzyme activities and reduced MDA) observed in both serum and liver across TM and TM+FA groups, TM+FA further significantly boosted SOD and GSH-Px activities specifically in the heart. This finding suggests a potential link between FA treatment and better cardiac health in broilers, which may be associated with HSP gene expression.

It has been shown that HSP expression in chickens is closely associated with ROS induced oxidative damage (Altan et al., 2003). Zhao et al. (2014) demonstrated that oxidative stress triggers HSP expression in avian immune organs, suggesting a potential protective role of HSP against oxidative damage. Research has extensively elucidated the roles of HSP70 and HSP90, two prominent members of the heat shock protein family, in mediating cell tolerance and regulating the cell cycle. Elevated temperatures trigger a cascade of cellular responses, including the upregulation of HSP synthesis. These proteins subsequently facilitate the production of other proteins, oversee their correct folding and translocation, and protect damaged proteins from oxidative breakdown and programmed cell death (Abare et al., 2023; Wadood et al., 2026). Through these mechanisms, HSPs significantly contribute to a cell's ability to recover following a stressful event. In this study, both TM and TM+FA significantly down-regulated the expression of *Bax* and *HSP90* genes in broilers. Furthermore, the TM+FA regimen more pronouncedly upregulated the expression of *HSP70* mRNA. These correlational findings point to a potential regulatory role of HSP90 in mediating the anti-apoptotic effects observed with both TM and TM+FA treatments. Concurrently, the enhanced cardiac antioxidant capacity in the TM+FA group appears to be associated with *HSP70* mRNA upregulation, although the direction of causality remains to be experimentally determined.

We have proposed two explanations for the mismatch between physiological indices and growth performance. To further clarify the effects of TM combined with FA, future research could focus on determining the most appropriate FA supplementation period during incubation to facilitate FA-mediated TM repair. Moreover, long-term outcomes regarding broiler performance and related epigenetic regulatory pathways should be further explored.

## Conclusion

Soaking TM-treated embryonic eggs in FA solution was associated with altered metabolism and growth of broilers at 42 days of age, concurrent with changes in hypothalamic *TRH* gene expression and serum hormone levels. TM alone usually impairs hatchability and healthy chick rate, while FA treatment partially reversed such adverse impacts and improved both hatchability and chick quality.This combined treatment was also correlated with reduced markers of cell apoptosis, altered HSP gene expression, and enhanced antioxidant capacity in broilers. These findings provide preliminary evidence that FA soaking of TM-treated embryonic eggs during embryogenesis links to improved thermotolerance-related indicators in broilers, suggesting embryonic FA supplementation could serve as an effective strategy to optimize TM application. However, the underlying regulatory mechanisms—particularly those involving TRH, HSP signaling, and antioxidant pathways—require further investigation through targeted mechanistic studies.

## Funding

This study was supported by the 10.13039/501100004610Science and Technology Support Program of Sichuan Province (Grant No. 2023NSFSC0236, completed), and the 10.13039/501100012226Fundamental Research Funds for the Central Universities, Southwest Minzu University (Grant No. ZYN2025096, ongoing).

## Author Contribution

Nawei Jike (Co-first author): Joined the whole animal work including egg incubation, broiler feeding, sample collection and all biochemical tests. The author took part in forming the research ideas, collated all test data, finished statistical analysis, made all statistical figures and arranged related image materials, wrote the full first draft of the paper, and revised the whole manuscript based on comments from editors and reviewers.Hanke Wang (Co-first author): Finished all animal experiments, including egg incubation, broiler raising, sample collection, as well as tests for physiological indicators and gene expression. The author shared ideas to improve the trial design, drew all charts and tables, helped organize test data, took part in writing the first draft, and polished the Results and Discussion parts.Shixiong Lai: Helped design part of the experiment plan, took part in sample collection, analyzed test data and explained the results we got, and revised the manuscript.Yucai Sun: Measured serum biochemical indicators, sorted original test data, drew statistical charts, put together all extra figures of this study, and revised relevant manuscript content.Yongliang Zhang: Analyzed all test results and HE stained tissue slices, wrote the first draft of Results and Discussion, and adjusted the wording of related paragraphs.Tingting Liu: Modified the Discussion section repeatedly, helped analyze all test results together, and checked and edited the full manuscript.Fengzhe Chen: Collected broiler samples at each scheduled sampling time, simply sorted all raw test data, and assisted in polishing manuscript text.Zhichao Hei: Processed collected samples for laboratory assays, checked all sorted data to eliminate errors, and edited part of the experimental methods section.Weidong Feng: Provided research resources for the study, and assisted in collecting and filing all original test data.Kaiqing Rao (Corresponding author): Proposed the overall research idea, designed the complete experimental scheme, supervised all animal experimental procedures, sourced research resources and provided financial support for this project, revised the full manuscript to strengthen core academic content, coordinated and revised all response letters addressing editorial and reviewer comments, and took full responsibility for this research project.

## Disclosures

The authors declare that there is no conflict of interest associated with the paper. The authors alone are responsible for the content and writing of this article.

## Appendix Supplementary materials

Image, application 1
